# Cholera Toxin: An Intracellular Journey into the Cytosol by Way of the Endoplasmic Reticulum

**DOI:** 10.3390/toxins2030310

**Published:** 2010-03-05

**Authors:** Naomi L. B. Wernick, Daniel J.-F. Chinnapen, Jin Ah Cho, Wayne I. Lencer

**Affiliations:** 1 GI Cell Biology, Children's Hospital (and Harvard Medical School), 300 Longwood Avenues, Enders 720, Boston, MA 02115, USA; Email: Naomi.Wernick@childrens.harvard.edu (N.L.B.W.); Dan.Chinnapen@childrens.harvard.edu (D.J.-F.C.); JinAh.Cho@childrens.harvard.edu (J.A.C.); 2 The Harvard Digestive Diseases Center, Boston, MA 02115, USA

**Keywords:** cholera toxin, retro-translocation, ERAD, retrograde pathway, lipid rafts

## Abstract

Cholera toxin (CT), an AB_5_-subunit toxin, enters host cells by binding the ganglioside GM1 at the plasma membrane (PM) and travels retrograde through the *trans*-Golgi Network into the endoplasmic reticulum (ER). In the ER, a portion of CT, the enzymatic A1-chain, is unfolded by protein disulfide isomerase and retro-translocated to the cytosol by hijacking components of the ER associated degradation pathway for misfolded proteins. After crossing the ER membrane, the A1-chain refolds in the cytosol and escapes rapid degradation by the proteasome to induce disease by ADP-ribosylating the large G-protein Gs and activating adenylyl cyclase. Here, we review the mechanisms of toxin trafficking by GM1 and retro-translocation of the A1-chain to the cytosol.

## 1. Introduction

**Figure 1:**
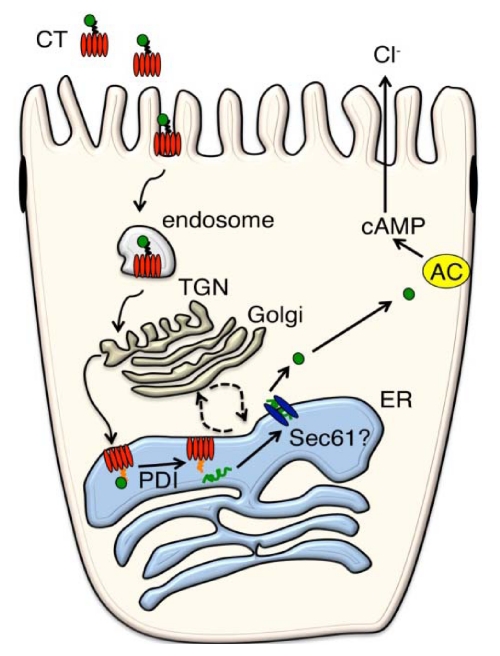
Current model for CT cell entry and intoxication. CT, *via* its B-subunit, binds to GM1 on the apical membrane of intestinal epithelial cells. It then traffics through early and recycling endosomes [2,3] to the TGN, perhaps bypassing the Golgi apparatus for eventual delivery to the ER [4,5]. From here, CT can recycle between the Golgi and ER (dotted arrows) [4]. Once inside the ER, the A1-chain is unfolded by PDI [1], recognized by the ER Hsp70 chaperone BiP (heavy chain binding protein) [6] and is presumably rendered a soluble substrate for retro-translocation by the ERAD-lumenal pathway [7]. Retro-translocation possibly involves the ER membrane proteins Hrd1, derlin-1 [8,9,10] and the Sec61 translocon [11]. Upon entry into the cytosol, the A1-chain refolds into its native conformation and activates adenylate cyclase (AC) by ADP-ribosylation of the hetero-trimeric G-protein Gs. The increase in cAMP causes chloride secretion and the massive diarrhea that typifies cholera [12].

Cholera toxin (CT) is the causative agent for the massive secretory diarrhea seen in Asiatic cholera. CT is secreted by the gram-negative bacterium *Vibrio cholerae* in the intestinal lumen. From here, it passes into and across the barrier of polarized epithelial cells that line the intestine by hijacking endogenous pathways to induce toxicity (Figure 1). CT begins its journey into the host cell by binding to the ganglioside GM1. The toxin then enters the cell by various modes of endocytosis, traffics retrograde from the plasma membrane (PM) to the *trans*-Golgi Network (TGN) and ultimately reaches the endoplasmic reticulum (ER). Trafficking in this pathway is likely dependent on the ability of GM1 to associate with lipid rafts. Once in the ER, CT masquerades as a terminally misfolded protein to co-opt mechanisms for ER associated degradation (ERAD) and cross the ER membrane into the cytosol in a process termed retro-translocation. In the lumen of the ER, the enzymatically active portion of the A-subunit, the A1-chain, is unfolded by protein disulfide isomerase (PDI) [1]. How the A1-chain crosses the ER membrane is unclear. Once in the cytosol, the enzymatically active A1-chain refolds, avoids rapid degradation, gains access to its substrate, and induces toxicity. This paper will review current ideas for how the toxin gains access to the ER and hijacks the ERAD pathway. (Table 1 summarizes the key host cell factors involved.)

## 2. Structure and Function

CT belongs to the larger family of AB toxins [13]. These toxins are characterized by having an enzymatically active A-domain, responsible for inducing toxicity, and a cell binding B-domain, responsible for cell entry. The AB toxins often consist of a single polypeptide chain that are cleaved into individual A and B components (e.g., ricin and diphtheria toxin), while others are comprised of individual A and B polypeptides that self assemble during the process of intoxication (e.g., anthrax toxin.) A subset of the AB family, the AB_5_ family of toxins, are comprised of six polypeptides, a single A-subunit and a homopentameric B-subunit that self assemble to form the holotoxin prior to secretion from the microbe. CT typifies this AB_5_ family of toxins. Other members include the closely related heat labile enterotoxins, as well as shiga toxin, the shiga-like toxins, and pertussis toxin.

**Figure 2:**
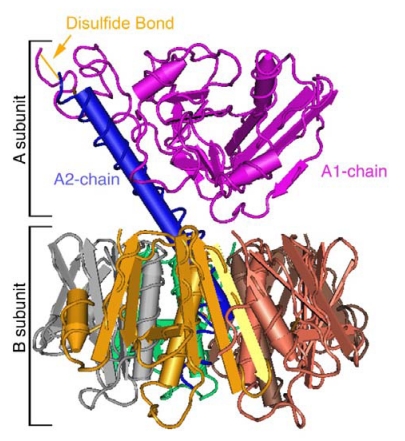
Three-dimensional structure of CT [14,15]. The A-subunit non-covalently associates with the pentameric B-subunit. The A-subunit is further subdivided into A1- and A2-chains, which are separated by a protease cleavage site and are joined by a disulfide bond and further non-covalent interactions.

The 27 kDa A-subunit of CT is comprised of an A2- and enzymatically active A1-chain, which is linked non-covalently to the B-subunit *via* the A2-chain (Figure 2). The A-subunit contains a serine-protease cleavage site located between residues 192 and 195 that allows for cleavage of the A-subunit into two polypeptides: the A2-chain and A1-chain. A disulfide bond between residues 187 and 199 bridges these chains together. Both the peptide and disulfide bonds must be broken before the A1-chain can enter the cytosol of host cells. The B-subunit consists of five 11.5 kDa peptides assembled non-covalently into a stable homopentamer that binds to the ganglioside GM1 on the PM. The B-subunit-GM1 complex carries the A-subunit into the ER [4]. 

Following retro-translocation, the A1-chain enters the cytosol as an active ADP-ribosyltransferase that modifies the heterotrimeric G protein, Gsα. Modification of this G protein leads to the constitutive activation of adenylate cyclase and the rapid production of cAMP. In intestinal cells, this induces intestinal chloride secretion, which is accompanied by a massive movement of water and the diarrhea that is the hallmark of cholera.

## 3. Retrograde from the PM to the ER

### 3.1. Binding and Entry *via* the PM

**Figure 3:**
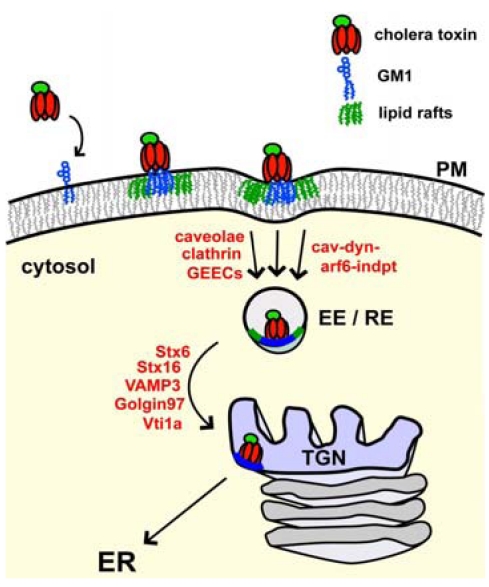
Retrograde Trafficking from the PM to the ER. CT binds to the ganglioside GM1 (blue) found in membrane microdomains (lipid rafts-green) on the plasma membrane of host cells, and can cluster five GM1 molecules at once. The toxin enters the cell by various endocytic mechanisms, including clathrin and caveolin-dependent, as well as caveolin and dynamin-independent mechanisms, and traffics to early and recycling endosomes [26,27,28,32]. Transport to the TGN involves many different proteins including V- and T-SNAREs [35,36]. From the TGN, the toxin traffics to the ER, apparently bypassing the Golgi-cisternae.A fraction of CT might be transported directly from endosome to ER. In the ER, the A1-chain is unfolded and retro-translocated to the cytosol.Available evidence indicates that the toxin B-subunit is bound to GM1 for the duration of the journey back to the ER.

Cholera toxin begins its intracellular journey by binding to the ganglioside GM1 located on the outer leaflet of apical membranes of intestinal epithelial cells. GM1 is the vehicle that transports the toxin all the way backwards in the secretory pathway from PM to ER. When clustered by binding to the B-subunit, GM1 shows preferential association with membrane microdomains, termed “lipid rafts,” composed of gangliosides, cholesterol, GPI-anchored proteins and sphingomyelin (Figure 3) [16,17]. Spatial and temporal information on these microdomain structures has proven difficult to obtain with conventional imaging methods due to resolution limits. Recent advances, however, have revealed they may be very small in size and highly dynamic, existing for 10-20 ms and having a diameter of less than 20-50 nm [18,19,20]. CT can bind up to five GM1 molecules at once by virtue of the ring-like pentameric structure of the B-subunit. This essentially clusters the ganglioside ceramide chains in the plane of the membrane and has been shown to increase the affinity of the GM1-CT complex with lipid rafts [16]. The association of CT with lipid rafts appears to be crucial for toxicity [4] and toxin clustering of GM1 likely increases the efficiency of retrograde trafficking to the ER, perhaps by stabilizing lipid raft microdomains, by inducing membrane curvature [21], or by simply enhancing the affinity for binding GM1 [22]. 

The heterogeneous lipid composition of the PM has been shown to be important for CT function. In cells depleted of membrane cholesterol or sphingomyelin, downstream trafficking to the Golgi is inhibited and toxicity is attenuated [23,24]. Similar observations have been made for Shiga-like toxin [25]. Presumably, this reflects the dependence of CT and Shiga-like toxin on lipid raft microdomains. 

### 3.2. Endocytosis and Trafficking Back to the ER

Toxin endocytosis en route to early endosomes from the PM can occur by various mechanisms [26,27,28]. Some pathways appear more or less prominent depending on the cell type studied. Early studies demonstrated a non-clathrin coated caveolar mechanism of endocytosis as the major route of entry in rat liver, HeLa, 3T3 and BHK cells [26,29,30]. Endocytosis has also been shown to occur *via* clathrin-mediated mechanisms in human intestinal Caco2 cells and pig enterocytes [27,31,32]. In enterocytes, caveolar endocytosis appeared to be a minor pathway. Non-clathrin/non-caveolar modes of endocytosis have also been observed with monkey kidney epithelial (BSC-1) cells and mouse embryonic fibroblasts (MEFs) [28,33]. Simultaneous dominant-negative expression of mutant proteins in BSC-1 cells to inhibit clathrin-, caveolar- and ARF6-dependent endocytosis revealed a non-dynamin dependent mechanism for vesicle budding. Immunoelectron microscopy of MEFs lacking caveolin-1 have identified tubular and ring-like endocytic carriers termed GPI-enriched early endosomal compartments (GEECs) that originate from the PM and are involved with cdc42-mediated uptake mechanisms [33]. The results of these studies show that CT can enter the cell by many different means. Regardless of the mode of entry, however, the toxin is found in early endosomal vesicles containing the small GTPase Rab5. Perhaps, the decisive steps in sorting retrograde to the ER take place in this compartment.

Early endosome to Golgi transport for CT is not completely understood, but the toxins, in general, are thought to move from early endosomes to TGN, independent of the late endosome pathway [34,35,36]. The mannose 6-phosphate receptor uses the late endosome to TGN route involving Rab9, T-SNARES Syntaxin 10 (STX10), STX16 and V-SNARES VAMP3, and Vti1a. CT and Shiga, however, are transported directly to the TGN from the early endosome *via* STX6-, Rab6A’-, and Golgin97-mediated vesicle docking to the TGN involving the aforementioned SNAREs, with exception of STX10 [36,37,38]. This pathway has been shown to be dependent on cdc42 and utilizes actin and microtubules (reviewed by Sandvig *et al.* [39]). Both CT and Shiga toxin require STX5, though Shiga appears to be more sensitive to depletion of this protein [35]. The retromer complex has also been identified as a key component in retrograde Shiga transport from endosomes to the TGN [40,41]. The retromer consists of sorting nexins SNX1, SNX2 and Vps26, Vps29 and Vps35, and functions by associating with membrane lipids in early and recycling endosomes to sense curvature and facilitate tubulation away from these organelles. Overall, it appears that Shiga and CT use a similar, though perhaps not identical, pathway from the PM to the TGN. 

From the TGN, cholera toxin is transported to the ER *via* a COPI-independent pathway in human intestinal T84 cells [5]. Using the small molecule agent, Exo2, which collapses the Golgi stacks but does not affect the TGN, it was found that CT-GM1 might bypass the Golgi cisternae trafficking directly to the ER [5]. The pathway from TGN to ER in almost all cells is, however, sensitive to Brefeldin A, a fungal metabolite known to induce tubulation of the TGN and subsequent dispersion into endosomes and ER membranes [42]. The brefeldin block occurs before toxin entry into the TGN. It is possible that a fraction of the toxin moves directly from endosomes to the ER, bypassing the TGN entirely (Saslowsky and Lencer unpublished data). Interestingly, the A-subunit, of CT harbors a conserved KDEL motif that is not essential for transport to the ER [43]. Rather, the KDEL-motif appears to allow for retention in the ER, by recycling CT between ER and *cis*-Golgi, and enhanced interaction with ERAD proteins for retro-translocation.

## 4. From ER to cytosol

### 4.1. The A1-chain Hijacks the ERAD Pathway

Proteolytic cleavage of the loop connecting the A1- and A2-chains of CT occurs following secretion from *Vibrio cholerae* in the intestinal lumen. The A and B-subunits, however, remain folded stably together; even after the disulfide bond linking the two chains is reduced [44]. The A1-chain must actively be released from the toxin complex. This is achieved within the ER lumen by the redox-dependent chaperone, protein disulfide isomerase (PDI) [1]. Upon entering the ER, the A-chain of CT (CTA) is recognized by the reduced form of PDI, which binds to and unfolds the A1-chain. At the same time, the PDI-like protein, Erp72, works in opposition to mediate the refolding and ER retention of CTA [45]. In the end, PDI succeeds in unfolding the A1-chain. The basis for this recognition is not known, but might be initiated by a relative instability of fold of the A-subunit [46,47,48], or perhaps by exposure of the hydrophobic C-terminal domain of the A1-chain upon unfolding by PDI, thus triggering the response for disposing of terminally-misfolded host cell proteins in the secretory pathway [49,50]. Recent NMR-based structural data as well as 4,4’-bis(1-anilinonaphthalene 8-sulfonate) fluorescence studies complement this hypothesis by illustrating disorder within the C-terminal A1_2_ and A1_3_ regions [51]. Nevertheless, the exact motif responsible for PDI binding remains unknown. The PDI-A1-chain complex is then presumably targeted to a protein on the lumenal membrane of the ER [52], after which the ER oxidase, Ero1, likely oxidizes PDI to induce the release of the A1-chain [52]. PDI may not be the only chaperone responsible for unfolding of the A1-chain. Additional *in vitro* evidence suggests that the Hsp70 chaperone BiP (heavy chain binding protein) plays a major role in maintaining the A1-chain in a soluble, export-competent state [6]. Bip allows for proper folding of nascent peptide chains in the secretory pathway and plays an important role in regulating the unfolded protein ER stress response and in the retro-translocation of several ERAD substrates [53].

### 4.2. Escape from the ER into the Cytosol

Many groups speculated that CT, like other ERAD substrates [54,55], crossed the ER membrane through a protein-conducting channel initially thought to be the translocon Sec61 [56,57]. Evidence for this idea was reported by Schmitz *et al.* [11]. These experiments were performed in a cell-free export system using translocation-competent microsomes. The results show that CT co-immunoprecipitates with Sec61β and that blockade of Sec61 by ribosome binding inhibits retro-translocation of the A1-chain. There are problems, however, with the idea that Sec61 operates in this way [50], and other possibilities for the identity of the channel have emerged [8,9,10], including the idea that a channel is not required at all [58]. 

We presume the PDI-A1-chain complex is released to the ER lumen when dissociated from the B-subunit. In yeast, misfolded ER lumenal proteins are processed for retro-translocation by a core multi-protein complex containing the Derlin-1 homologue Der1 and the ubiquitin E3 ligase Hrd1 (the ERAD-L pathway) [7,59]. In mammalian cells, the pathways for retro-translocation are more diversified [55,60], but the Derlin and Hrd1/gp78 proteins are required for retro-translocation of all lumenal ERAD substrates so far tested [61,62]. Hrd1 and Derlin-1 are multi-spanning membrane proteins, both considered candidates for the protein-conducting channel responsible for retro-translocation of ER lumenal or membrane substrates [55]. Most (but not all) ERAD substrates are ubiquitinated, primarily on Lys residues, during the retro-translocation reaction, and both Hrd1 and gp78 are E3-ligases known to play a role in this process [63].

Recently, Dixit *et al.* and Bernardi *et al.* have implicated a role for the ER membrane proteins Derlin-1 and Hrd1 in retro-translocation of the CT A1-chain [8,9,10]. Dixit *et al.* [8] showed that cells upregulated in the expression of Derlin-1 protein were more sensitive to intoxication by CT, and cells lacking Derlin-1 (by siRNA suppression) were resistant. Interaction between Derlin-1 and CT was supported by co-immunoprecipitation. Bernardi *et al.* found a role for Derlin-1 in the retro-translocation of CT using 293T cells transiently expressing dominant negative Derlin-1 [9]. Here, retro-translocation of the CT A1-chain was measured directly, and less A1-chain was found in the cytosol of cells expressing dominant negative Derlin-1. Toxicity was also inhibited. Again, direct interaction between the two proteins was suggested by co-immunoprecipitation.

Bernardi *et al.* [10] also showed a role for Hrd1 and another E3 ubiquitin ligase, gp78, both known to interact with Derlin-1 [64,65] and implicated in the retro-translocation of terminally misfolded (or otherwise targeted) lumenal (soluble) and membrane protein ERAD-substrates [7,66,67]. Hrd1 functions as an oligomer in complex with USA1 [68], as a homodimer [69] and as a heterodimer in complex with gp78 [65]. Dominant negative Hrd1 as well as siRNA to suppress Hrd1 were used in a cell-based retro-translocation assay, and both were found to inhibit movement of CT from ER to cytosol. Hrd1 was found to co-immunoprecipitate with CT except when dominant negative Derlin-1 was over-expressed, suggesting that CT might interact with Derlin-1 before Hrd1. Dominant negative gp78 also inhibited retro-translocation of the A1-chain, and like Hrd1, gp78 was found to co-immunoprecipitate with CT. Because Hrd1 and gp78 are E3 ubiquitin ligases, this paper also studied the role of the E2 ubiquitin-conjugating enzyme, Ube2g2, and found it to be involved. In some ways, these results were not expected because the A1-chain, unlike most ERAD substrates [55], does not depend on the presence of any of the three available primary amines for retro-translocation [70]. Perhaps ubiquitination of other cellular components, rather than direct ubiquitination of CT itself is required [10]. 

The model emerging from these studies proposes that the CT-GM1 complex first interacts with Derlin-1 on the lumenal side of the ER membrane [8,9,10], where PDI first binds the A1-chain [1]. Derlin-1 then facilitates transfer of the A1-chain to Hrd1, and/or Sec61, after release from PDI [10,11]. Our group has recently found, however, that Derlin-1 and -2 are likely dispensable for retro-translocation of the A1-chain in zebrafish and mammalian cells (Saslowsky and Lencer unpublished), and exactly how the A1-chain interacts with Hrd1 or Sec61 to cross the ER membrane remains unclear. 

### 4.3. Cytosolic Factors

Endogenous ERAD substrates exit the ER and enter the cytosol for ubiquitination and degradation by the proteasome. CT [70] and the other AB toxins, however, escape this fate and are not rapidly degraded after retro-translocation, perhaps due to a paucity of lysine residues in their primary structures [49]. We have recently found, however, that a mutant form of the A1-chain undergoes retro-translocation but is rapidly degraded by the proteasome after being ubiquitinated at the two lysine residues present in the native toxin [71]. These results imply that other factors must be involved. It is possible that cytosolic chaperones or rapid refolding of the A1-chain upon entry into the cytosol allows CT to evade the ubiquitination machinery [71]. 

In a few cases, proteins not conjugated to ubiquitin are degraded by the proteasome [72]; but the majority of targets for proteasomal degradation are ubiquitinated. For many of these ubiquitinated ERAD substrates, interaction with the cytosolic AAA-ATPase p97 provides the driving force for retro-translocation [73,74,75]. One exception is the ubiquitin-independent ERAD substrate, pre-pro-alpha factor [76], which shows a lack of dependence on p97 in yeast [77]. Likewise, the bulk of evidence shows that p97 is dispensable for CT action in mammalian cells [9,78]. One study shows that over-expression of dominant negative p97 can inhibit CT-induced toxicity [80], but this result was not reproduced in our cell models [79]. McConnell *et al.*, in separate studies using RNAi to suppress p97, also found no effect on CT-induced toxicity [78]. Perhaps p97 binds the A1-chain as it emerges from the ER, but this does not appear to be essential. 

The lack of dependence on p97 [78,79], and the ability of the A1-chain to spontaneously refold when released from PDI *in vitro* [70,81], caused us to speculate that rapid refolding of the A1-chain as it emerged from the ER into the cytosol might act as a molecular ratchet to provide the driving force for retro-translocation [70]. Circular dichroism and fluorescence spectroscopy studies, however, show that the A1-chain is thermally unstable [48] and not likely to refold spontaneously *in vivo*. NMR as well as 4,4’-bis(1-anilinonaphthalene 8-sulfonate) fluorescence studies also demonstrate the partially unfolded state of the A1-chain *in vitro* [51]. Such instability challenges the idea of self-folding as a molecular ratchet and is proposed to explain why the A1-chain can be slowly degraded by the 20S proteasome *in vivo*, even in the absence of poly-ubiquitination [48,82]. Another important idea emerging from these studies is that the A1-chain might require binding to a cytosolic chaperone or other CT binding protein to stabilize its native conformation. *In vitro*, the active guanosine 5’-triphosphate (GTP) bound form of the eukaryotic ADP ribosylation factor 6 (ARF6), a cofactor for CT enzymatic activity *in vivo*, was found to do just that; it stabilized the A1-chain and protected it from degradation by the 20S proteasome [48,51]. Thus, binding of ARF6-GTP to the A1-chain restores order to the toxin *in vitro*, and this might explain how the toxin avoids rapid degradation (as discussed above) and induces toxicity *in vivo*. 

**Figure 4:**
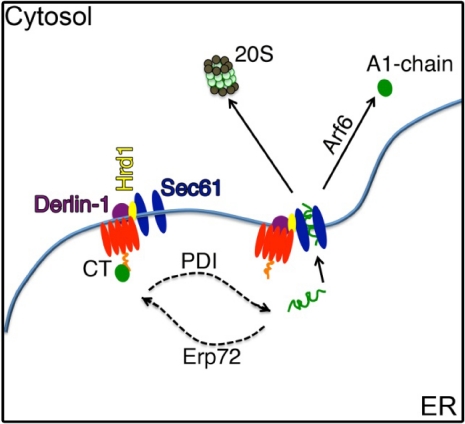
Schematic of CT retro-translocation. It is still unclear exactly how retro-translocation proceeds. This figure shows the candidate proteins thought to be involved. Following entry into the ER, CT remains bound to GM1 and can interact with Derlin-1 [8,9] and Hrd1 [10]. PDI unfolds CT [1], while Erp72 works in opposition to maintain it in a folded conformation [45]. There is evidence that Sec61 is the retro-translocation channel [11], but these studies are not fully conclusive and Hrd1 and gp78 are also candidates [10] for forming the protein-conducting channel. Perhaps, Derlin-1, Hrd1, and Sec61 are located in close proximity and act together. Finally, upon entry into the cytosol the A1-chain might immediately bind Arf6 or another chaperone to refold rapidly into a stable enzymatically active conformation and to avoid rapid degradation by the 20S proteasome [48,51].

A summary for how retro-translocation might progress is shown (Figure 4). Presumably, the last step in toxin action is a diffusion-limited reaction in the cytosol, where the A1-chain catalyzes the ADP-ribosylation of Gs to activate adenylyl cyclase. 

## 5. Summary

CT binds the ganglioside GM1 and co-opts a lipid-based sorting pathway to traffic retrograde from the PM into the ER where a portion of the toxin, the A1-chain, retro-translocates to the cytosol. Unlike most retro-translocation substrates, the A1-chain escapes degradation by the proteasome and refolds in the cytosol to induce disease. The mechanisms and host proteins involved (Table 1) in these diverse processes are fundamental to eukaryotic cell biology. They are just beginning to be understood in molecular detail, and much more is still to be discovered. 

**Table 1:** Host proteins likely involved in CT trafficking and A1-chain retro-translocation.

Host factor	Site of Action	Cellular Function
*Trafficking factors*		
Ganglioside GM1	Host cell membranes: PM, endosomes, Golgi, ER	Lipid receptor for toxin binding and trafficking
Lipid rafts	Host cell membranes	Putative small dynamic membrane microdomains that self assemble by phase separation of membrane lipids to form structures with functions in trafficking and signal transduction
Clathrin	PM, endosome, Golgi	Protein coat for some forms of endocytosis and vesicle budding
ARF 1- 6	PM, endosome, Golgi	Small GTPases involved in coat formation and membrane traffic. The ARF family was discovered by their ability to act as co-factors for the ADP-ribosylation activity of the CT A1-chain
Syntaxin 6 and 16	early endosome	Component of protein complex involved in fusion of vesicles moving from early endosome to TGN
Golgin97	TGN	Tethering factor for vesicles moving from early endosome to TGN
Retromer	endosome	Complex of proteins involved in transport of vesicles form early endosome to TGN
Rab 6A'	endosome, TGN	Small GTPases involved in sorting vesicles retrograde from early endosome and TGN to ER
VAMP3, Vti1a	TGN	Components of protein complex involved in fusion of vesicles moving from early endosome to TGN
SNX1 and 2	early/recycling endosome	Retromer components that contain phosphatidyl inositol binding and membrane curvature sensing BAR domains.
Vps26, 29 and 35	early/recycling endosome	Retromer components important for cargo selection, such as Shiga toxin and the mannose-6-phosphate receptor
*ERAD factors*		
Protein Disulfide Isomerase (PDI)	ER lumen	Disulfide bond isomerase and protein chaperone, unfolds and dissociates the A1 chain for the B-subunit
ER Protein 72 (Erp72)	ER lumen	PDI-like molecule with counteracting function to refold the A1-chain
ER oxidase 1 (Ero1)	ER lumen	ER oxidase that oxidizes PDI to release the A1-chain
Heavy chain binding protein (BiP)	ER lumen	HSP70 chaperone with major functions in protein folding and ERAD
Sec 61 translocon	ER membrane	Core component of the translocon that ribosomes dock with to allow for translocation of membrane and secreted proteins into the ER during biosynthesis. It is also a candidate for the protein conducting channel in ERAD.
Derlin-1	ER membrane	Component of the core Hrd1 complex required for retro-translocation of lumenal ERAD substrates. It is also a candidate for the protein conducting channel in ERAD.
Hrd1	ER membrane	ER membrane ubiquitin E3 ligase forming central component of a protein complex involved in retro-translocation of ER lumenal and membrane ERAD substrates. It is also a candidate for the protein conducting channel in ERAD.
gp78	ER membrane	ER membrane ubiquitin E3 ligase forming central component of a protein complex involved in retro-translocation of ER lumenal and membrane ERAD substrates.
Ubiquitin-conjugating enzyme (Ube)	Cytosol	Ubiquitin E2 ligase: Enzyme required before the E3 ligases in the pathway of conjugating ubiquitin to primarily lysine residues on proteins.
Ube2g2	Cytosol, ER associated	Ubiquitin E2 ligase: Enzyme required before the E3 ligases in the pathway of conjugating ubiquitin to primarily lysine residues on proteins.
AAA-ATPase p97	Cytosol	AAA-ATPase involved in chaperone function, proteasomal degradation. It is key for retro-translocation of most ERAD substrates, perhaps providing the driving force for the retro-translocation reaction itself. However, it is not required for retro-translocation of the CT A1-chain.

## References

[1] Tsai B., Rodighiero C., Lencer W.I., Rapoport T. (2001). Protein disulfide isomerase acts as a redox-dependent chaperone to unfold cholera toxin. Cell.

[2] Nichols B.J., Kenworthy A.K., Polishchuk R.S., Lodge R., Roberts T.H., Hirschberg K., Phair R.D., Lippincott-Schwartz J. (2001). Rapid cycling of lipid raft markers between the cell surface and Golgi complex. J. Cell Biol..

[3] Richards A.A., Stang E., Pepperkok R., Parton R.G. (2002). Inhibitors of COP-mediated Transport and Cholera Toxin Action Inhibit Simian Virus 40 Infection. Mol. Biol. Cell.

[4] Fujinaga Y., Wolf A.A., Rodigherio C., Wheeler H., Tsai B., Allen L., Jobling M.G., Rapoport T., Holmes R.K., Lencer W.I. (2003). Gangliosides that associate with lipid rafts mediate transport of cholera toxin from the plasma membrane to the ER. Mol. Biol. Cell.

[5] Feng Y., Jadhav A.P., Rodighiero C., Fujinaga Y., Kirchhausen T., Lencer W.I. (2004). Retrograde transport of cholera toxin from the plasma membrane to the endoplasmic reticulum requires the trans-Golgi network but not the Golgi apparatus in Exo2-treated cells. EMBO Rep..

[6] Winkeler A., Godderz D., Herzog V., Schmitz A. (2003). BiP-dependent export of cholera toxin from endoplasmic reticulum-derived microsomes. FEBS Lett..

[7] Carvalho P., Goder V., Rapoport T.A. (2006). Distinct ubiquitin-ligase complexes define convergent pathways for the degradation of ER proteins. Cell.

[8] Dixit G., Mikoryak C., Hayslett T., Bhat A., Draper R.K. (2008). Cholera toxin up-regulates endoplasmic reticulum proteins that correlate with sensitivity to the toxin. Exp. Biol. Med. (Maywood).

[9] Bernardi K.M., Forster M.L., Lencer W.I., Tsai B. (2008). Derlin-1 facilitates the retro-translocation of cholera toxin. Mol. Biol. Cell.

[10] Bernardi K.M., Williams J.M., Kikkert M., van Voorden S., Wiertz E.J., Ye Y., Tsai B. (2010). The E3 ubiquitin ligases hrd1 and gp78 bind to and promote cholera toxin retro-translocation. Mol. Biol. Cell.

[11] Schmitz A., Herrgen H., Winkeler A., Herzog V. (2000). Cholera toxin is exported from microsomes by the sec61p complex. J. Cell Biol..

[12] Spangler B.D. (1992). Structure and function of cholera toxin and the related *Escherichia coli* heat-labile enterotoxin. Microb Rev..

[13] Falnes P.O., Sandvig K. (2000). Penetration of protein toxins into cells. Curr. Opin. Cell Biol..

[14] Sixma T.K., Pronk S.E., Kalk K.H., Wartna E.S., van Zanten B.A., Witholt B., Hol W.G. (1991). Crystal structure of a cholera toxin-related heat-labile enterotoxin from E.coli. Nature.

[15] Zhang R.G., Scott D.L., Westbrook M.L., Nance S., Spangler B.D., Shipley G.G., Westbrook E.M. (1995). The three-dimensional crystal structure of cholera toxin. J. Mol. Biol..

[16] Panasiewicz M., Domek H., Hoser G., Kawalec M., Pacuszka T. (2003). Structure of the ceramide moiety of GM1 ganglioside determines its occurrence in different detergent-resistant membrane domains in HL-60 cells. Biochemistry.

[17] Kaiser H.J., Lingwood D., Levental I., Sampaio J.L., Kalvodova L., Rajendran L., Simons K. (2009). Order of lipid phases in model and plasma membranes. Proc. Natl. Acad. Sci. USA.

[18] Cicuta P., Keller S.L., Veatch S.L. (2007). Diffusion of liquid domains in lipid bilayer membranes. J. Phys. Chem. B.

[19] Sharma P., Varma R., Sarasij R.C., Ira, Gousset K., Krishnamoorthy G., Rao M., Mayor S. (2004). Nanoscale organization of multiple GPI-anchored proteins in living cell membranes. Cell.

[20] Eggeling C., Ringemann C., Medda R., Schwarzmann G., Sandhoff K., Polyakova S., Belov V.N., Hein B., von Middendorff C., Schonle A., Hell S.W. (2009). Direct observation of the nanoscale dynamics of membrane lipids in a living cell. Nature.

[21] Ewers H., Romer W., Smith A.E., Bacia K., Dmitrieff S., Chai W., Mancini R., Kartenbeck J., Chambon V., Berland L., Oppenheim A., Schwarzmann G., Feizi T., Schwille P., Sens P., Helenius A., Johannes L. (2010). GM1 structure determines SV40-induced membrane invagination and infection. Nat. Cell Biol..

[22] Wolf A.A., Jobling M.G., Saslowsky D.E., Kern E., Drake K.R., Kenworthy A.K., Holmes R.K., Lencer W.I. (2008). Attenuated endocytosis and toxicity of a mutant cholera toxin with decreased ability to cluster gm1. Infect Immun..

[23] Saslowsky D.E., Lencer W.I. (2008). Conversion of apical plasma membrane sphingomyelin to ceramide attenuates the intoxication of host cells by cholera toxin. Cell Microbiol..

[24] Wolf A.A., Fujinaga Y., Lencer W.I. (2002). Uncoupling of the cholera toxin-G(M1) ganglioside receptor complex from endocytosis, retrograde Golgi trafficking, and downstream signal transduction by depletion of membrane cholesterol. J. Biol. Chem..

[25] Smith D.C., Sillence D.J., Falguieres T., Jarvis R.M., Johannes L., Lord J.M., Platt F.M., Roberts L.M. (2006). The association of shiga-like toxin with detergent-resistant membranes is modulated by glucosylceramide and is an essential requirement in the endoplasmic reticulum for a cytotoxic effect. Mol. Biol. Cell.

[26] Orlandi P.A., Fishman P.H. (1998). Filipin-dependent inhibition of cholera toxin: evidence for toxin internalization and activation through caveolae-like domains. J. Cell Biol..

[27] Torgersen M.L., Skretting G., van Deurs B., Sandvig K. (2001). Internalization of cholera toxin by different endocytic mechanisms. J. Cell Sci..

[28] Massol R.H., Larsen J.E., Fujinaga Y., Lencer W.I., Kirchhausen T. (2004). Cholera toxin toxicity does not require functional Arf6- and dynamin-dependent endocytic pathways. Mol. Biol. Cell.

[29] Montesano R., Roth J., Robert A., Orci L. (1982). Non-coated membrane invaginations are involved in binding and internalization of cholera and tetanus toxins. Nature.

[30] Tran D., Carpentier J.L., Sawano F., Gorden P., Orci L. (1987). Ligands internalized through coated or noncoated invaginations follow a common intracellular pathway. Proc. Natl. Acad. Sci. USA.

[31] Singh R.D., Puri V., Valiyaveettil J.T., Marks D.L., Bittman R., Pagano R.E. (2003). Selective caveolin-1-dependent endocytosis of glycosphingolipids. Mol. Biol. Cell.

[32] Hansen G.H., Dalskov S.M., Rasmussen C.R., Immerdal L., Niels-Christiansen L.L., Danielsen E.M. (2005). Cholera toxin entry into pig enterocytes occurs *via* lipid raft- and clathrin-dependent mechanism. Biochemistry.

[33] Kirkham M., Fujita A., Chadda R., Nixon S.J., Kurzchalia T.V., Sharma D.K., Pagano R.E., Hancock J.F., Mayor S., Parton R.G. (2005). Ultrastructural identification of uncoated caveolin-independent early endocytic vehicles. J. Cell Biol..

[34] Mallard F., Tang B.L., Galli T., Tenza D., Saint-Pol A., Yue X., Antony C., Hong W., Goud B., Johannes L. (2002). Early/recycling endosomes-to-TGN transport involves two SNARE complexes and a Rab6 isoform. J. Cell Biol..

[35] Amessou M., Fradagrada A., Falguieres T., Lord J.M., Smith D.C., Roberts L.M., Lamaze C., Johannes L. (2007). Syntaxin 16 and syntaxin 5 are required for efficient retrograde transport of several exogenous and endogenous cargo proteins. J. Cell Sci..

[36] Ganley I.G., Espinosa E., Pfeffer S.R. (2008). A syntaxin 10-SNARE complex distinguishes two distinct transport routes from endosomes to the trans-Golgi in human cells. J. Cell Biol..

[37] Del Nery E., Miserey-Lenkei S., Falguieres T., Nizak C., Johannes L., Perez F., Goud B. (2006). Rab6A and Rab6A' GTPases play non-overlapping roles in membrane trafficking. Traffic.

[38] Lu L., Tai G., Hong W. (2004). Autoantigen Golgin-97, an effector of Arl1 GTPase, participates in traffic from the endosome to the trans-golgi network. Mol. Biol. Cell.

[39] Sandvig K., Spilsberg B., Lauvrak S.U., Torgersen M.L., Iversen T.G., van Deurs B. (2004). Pathways followed by protein toxins into cells. Int. J. Med. Microbiol.

[40] Popoff V., Mardones G.A., Bai S.K., Chambon V., Tenza D., Burgos P.V., Shi A., Benaroch P., Urbe S., Lamaze C., Grant B.D., Raposo G., Johannes L. (2009). Analysis of articulation between clathrin and retromer in retrograde sorting on early endosomes. Traffic.

[41] Popoff V., Mardones G.A., Tenza D., Rojas R., Lamaze C., Bonifacino J.S., Raposo G., Johannes L. (2007). The retromer complex and clathrin define an early endosomal retrograde exit site. J. Cell Sci..

[42] Nambiar M.P., Oda T., Chen C., Kuwazuru Y., Wu H.C. (1993). Involvement of the Golgi region in the intracellular trafficking of cholera toxin. J. Cell Physiol..

[43] Lencer W.I., Constable C., Moe S., Jobling M., Webb H.M., Ruston S., Madara J.L., Hirst T., Holmes R. (1995). Targeting of cholera toxin and *E. coli* heat labile toxin in polarized epithelia: role of C-terminal KDEL. J. Cell Biol..

[44] Mekalanos J.J., Collier R.J., Romig W.R. (1979). Enzymic activity of cholera toxin. II. Relationships to proteolytic processing, disulfide bond reduction, and subunit composition. J. Biol. Chem..

[45] Forster M.L., Sivick K., Park Y.N., Arvan P., Lencer W.I., Tsai B. (2006). Protein disulfide isomerase-like proteins play opposing roles during retrotranslocation. J. Cell Biol..

[46] Goins B., Freire E. (1988). Thermal stability and intersubunit interactions of cholera toxin in solution and in association with its cell-surface receptor ganglioside G_M1_. Biochem..

[47] Surewicz W.K., Leddy J.J., Mantsch H.H. (1990). Structure, stability, and receptor interaction of cholera toxin as studied by Fourier-transform infrared spectroscopy. Biochemistry.

[48] Pande A.H., Scaglione P., Taylor M., Nemec K.N., Tuthill S., Moe D., Holmes R.K., Tatulian S.A., Teter K. (2007). Conformational instability of the cholera toxin A1 polypeptide. J. Mol. Biol..

[49] Hazes B., Read R.J. (1997). Accumulating evidence suggests that several AB-toxins subvert the endoplasmic reticulum-associated protein degradation pathway to enter target cells. Biochemistry.

[50] Tsai B., Rapoport T. (2002). Retro-translocation of proteins from the endoplasmic reticulum into the cytosol. Nature Rev. Cell Biol..

[51] Ampapathi R.S., Creath A.L., Lou D.I., Craft J.W., Blanke S.R., Legge G.B. (2008). Order-Disorder-Order Transitions Mediate the Activation of Cholera Toxin. J. Mol. Biol..

[52] Tsai B., Rapoport T. (2002). Unfolded cholera toxin is transferred to the ER membrane and released from protein disulfide isomerase upon oxidation by Ero1. J. Cell Biol..

[53] Nishikawa S., Brodsky J.L., Nakatsukasa K. (2005). Roles of molecular chaperones in endoplasmic reticulum (ER) quality control and ER-associated degradation (ERAD). J. Biol. Chem..

[54] Brodsky J.L., McCracken A.A. (1999). ER protein quality control and proteasome-mediated protein degradation. Semin. Cell Dev. Biol..

[55] Vembar S.S., Brodsky J.L. (2008). One step at a time: endoplasmic reticulum-associated degradation. Nat. Rev. Mol. Cell Biol..

[56] Wiertz E.J., Tortorella D., Bogyo M., Yu J., Mothes W., Jones T.R., Rapoport T.A., Ploegh H.L. (1996). Sec61-mediated transfer of a membrane protein from the endoplasmic reticulum to the proteasome for destruction. Nature.

[57] Pilon M., Schekman R., Romisch K. (1997). Sec61p mediates export of a misfolded secretory protein from the nedoplasmic reticulum to the cytososl for degradation. EMBO J..

[58] Ploegh H.L. (2007). A lipid-based model for the creation of an escape hatch from the endoplasmic reticulum. Nature.

[59] Denic V., Quan E.M., Weissman J.S. (2006). A luminal surveillance complex that selects misfolded glycoproteins for ER-associated degradation. Cell.

[60] Brodsky J.L., Wojcikiewicz R.J. (2009). Substrate-specific mediators of ER associated degradation (ERAD). Curr. Opin. Cell Biol..

[61] Oda Y., Okada T., Yoshida H., Kaufman R.J., Nagata K., Mori K. (2006). Derlin-2 and Derlin-3 are regulated by the mammalian unfolded protein response and are required for ER-associated degradation. J. Cell Biol..

[62] Okuda-Shimizu Y., Hendershot L.M. (2007). Characterization of an ERAD pathway for nonglycosylated BiP substrates, which require Herp. Mol. Cell.

[63] Meusser B., Hirsch C., Jarosch E., Sommer T. (2005). ERAD: the long road to destruction. Nat. Cell Biol..

[64] Lilley B.N., Ploegh H.L. (2005). Multiprotein complexes that link dislocation, ubiquitination, and extraction of misfolded proteins from the endoplasmic reticulum membran. Proc. Natl. Acad. Sci. USA.

[65] Ye Y., Shibata Y., Kikkert M., van Voorden S., Wiertz E., Rapoport T. A. (2005). Recruitment of the p97 ATPase and ubiquitin ligases to the site of retrotranslocation at the endoplasmic reticumul membrane. Proc. Natl. Acad. Sci. USA.

[66] Chen B., Mariano J., Tsai Y.C., Chan A.H., Cohen M., Weissman A.M. (2006). The activity of a human endoplasmic reticulum-associated degradation E3, gp78, requires its Cue domain, RING finger, and an E2-binding site. Proc. Natl. Acad. Sci. USA.

[67] Hirsch C., Gauss R., Horn S.C., Neuber O., Sommer T. (2009). The ubiquitylation machinery of the endoplasmic reticulum. Nature.

[68] Horn S.C., Hanna J., Hirsch C., Volkwein C., Schutz A., Heinemann U., Sommer T., Jarosch E. (2009). Usa1 functions as a scaffold of the HRD-ubiquitin ligase. Mol. Cell.

[69] Schulze A., Standera S., Buerger E., Kikkert M., van Voorden S., Wiertz E., Koning F., Kloetzel P.M., Seeger M. (2005). The ubiquitin-domain protein HERP forms a complex with components of the endoplasmic reticulum associated degradation pathway. J. Mol. Biol..

[70] Rodighiero C., Tsai B., Rapoport T.A., Lencer W.I. (2002). Role of ubiquitination in retro-translocation of cholera toxin and escape of cytosolic degradation. EMBO Rep..

[71] Wernick N.L., De Luca H., Kam W.R., Lencer W.I. (1990). N-terminal extension of the cholera toxin A1-chain causes rapid degradation after retro-translocation from ER to cytosol. J. Biol. Chem..

[72] Jariel-Encontre I., Bossis G., Piechaczyk M. Ubiquitin-independent degradation of proteins by the proteasome. Biochim. Biophys. Acta.

[73] Flierman D., Ye Y., Dai M., Chau V., Rapoport T.A. (2003). Polyubiquitin serves as a recognition signal, rather than a ratcheting molecule, during retrotranslocation of proteins across the endoplasmic reticulum membrane. J. Biol. Chem..

[74] Ye Y., Meyer H.H., Rapoport T.A. (2001). The AAA ATPase Cdc48/p97 and its partners transport proteins from the ER into the cytosol. Nature.

[75] Ye Y., Meyer H.H., Rapoport T.A. (2003). Function of the p97-Ufd1-Npl4 complex in retrotranslocation from the ER to the cytosol: dual recognition of nonubiquitinated polypeptide segments and polyubiquitin chains. J. Cell Biol..

[76] Werner E.D., Brodsky J.L., McCracken A.A. (1996). Proteasome-dependent endoplasmic reticulum-associated protein degradation: an unconventional route to a familiar fate. Proc. Natl. Acad. Sci. USA.

[77] Lee R.J., Liu C.W., Harty C., McCracken A.A., Latterich M., Romisch K., DeMartino G.N., Thomas P.J., Brodsky J.L. (2004). Uncoupling retro-translocation and degradation in the ER-associated degradation of a soluble protein. EMBO J..

[78] McConnell E., Lass A., Wojcik C. (2007). Ufd1-Npl4 is a negative regulator of cholera toxin retrotranslocation. Biochem. Biophys. Res. Commun..

[79] Kothe M., Ye Y., Wagner J.S., De Luca H.E., Kern E., Rapoport T.A., Lencer W.I. (2005). Role of p97 AAA-ATPase in the retrotranslocation of the cholera toxin A1 chain, a non-ubiquitinated substrate. J. Biol. Chem..

[80] Abujarour R.J., Dalal S., Hanson P.I., Draper R.K. (2005). p97 is in a complex with cholera toxin and influences the transport of cholera toxin and related toxins to the cytoplasm. J. Biol. Chem..

[81] Lencer W.I., Tsai B. (2003). The intracellular voyage of cholera toxin: going retro. Trends Biochem. Sci..

[82] Teter K., Allyn R.L., Jobling M.G., Holmes R.K. (2002). Transfer of the cholera toxin A1 polypeptide from the endoplasmic reticulum to the cytosol is a rapid process facilitated by the endoplasmic reticulum-associated degradation pathway. Infect Immun..

